# 

**Published:** 1906-09-15

**Authors:** 


### ANNOUNCEMENTS.

GRADUATES IN 1906.—The following list of dental colleges will show the number graduated by each school during the year 1906:

| | |
|---|---|
| Baltimore College of Dental Surgery | 45 |
| Pennsylvania College of Dental Surgery | 55 |
| Philadelphia Dental College | 48 |
| New York College of Dentistry | 41 |
| New York College of Dental and Oral Surgery | 40 |
| University of Michigan, College of Dental Surgery | 25 |
| University of Iowa, College of Dentistry | 20 |
| Chicago College of Dental Surgery | 78 |
| University of Pennsylvania, Dental Department | 112 |
| Ohio College of Dental Surgery | 29 |
| University of California, College of Dentistry | 24 |
| Kansas City Dental College | 27 |
| Washington University Dental Department | 27 |
| Vanderbilt University, Dental Department | 28 |
| Indiana Dental College | 22 |
| Northwestern University Dental School | 96 |
| University of Tennessee, Department of Dentistry | 8 |
| Maharry Medical College, School of Dentistry | 14 |
| Southern Dental College | 31 |
| Louisville College of Dentistry | 40 |
| University of Maryland, Dental Department | 42 |
| Royal College of Dental Surgeons, of Ontario | 49 |
| University of Minnesota, College of Dentistry | 41 |
| Western Reserve University, Dental Department | 13 |
| Detroit Medical College, Dental Department | 21 |
| Western Dental College | 39 |
| University of Buffalo, Dental Department | 34 |
| Richmond, Va., University, College of Medicine, Dental Department | 8 |
| Birmingham Dental College | 4 |
| Atlanta Dental College | 47 |

| | |
|---|---|
| Cincinnati College of Dental Surgery | 7 |
| Harvard University, Dental Department | 11 |
| St. Louis Dental College | 29 |
| Ohio Medical University, Dental Department | 20 |
| Baltimore Medical College, Dental Department | 17 |
| Milwaukee Medical College, Dental Department | 9 |
| North Pacific Dental College | 33 |
| University of Omaha | 15 |
| Colorado College of Dental Surgery | 13 |
| Pittsburg Dental College | 28 |
| San Francisco College of Physicians and Surgeons | 29 |
| Southern California University, Dental Department | 27 |
| University of Illinois, School of Dentistry | 43 |
| Georgetown University | 6 |
| New Orleans College of Dentistry | 24 |
| Keokuk Dental College | 8 |
| Wisconsin College of Physicians and Surgeons, DentalDepartment | 2 |
| Lincoln, Nebraska, Dental College | 7 |
| Texas State Dental College | 5 |
| | Total, 1,441 |

Last year there were 2,627 graduates, or 1,186 more than this year. This shows how many students were kept out of our dental colleges by the adoption of the four year course of study. It amounts to nearly 50 percent.

—✦—

ST. LOUIS DENTAL SOCIETY CELEBRATES WITH A GOLDEN JUBILEE.—The St. Louis Dental Society, will celebrate its Golden Anniversary with a Banquet and Entertainment sometime in November, when the Fiftieth Year of its existence will be completed. Eminent men in the profession outside of the city and state will participate.

More detailed anouncement will be given in October issue.

Yours respectfully,

J. B. NEWBY,

*Chairman Committee on Arrangements.*

—✦—

COLUMBUS DENTAL LIBRARY.—The Columbus dentists held an enthusiastic meeting August 11th, at which it was decided that they would immediately organize and raise a sufficient fund to place a first-class dental library in the new Carnegie Library, which is nearing completion in that city. The building is a magnificent one, and the management is offering the dentists every possible inducement to develope their library. Any one who has old journals or text books that are rare will find a good secure depository for them, where they will be well cared for, and where they can be freely consulted by any one who may be interested. The dental profession of Ohio ought to take up this matter, as they can have one of the best libraries in the world at their command, if they see fit. Gifts of money, as well as books, will be accepted and properly credited. Communicate with Dr. E. C. Mills or Dr. Wm. H. Todd, Columbus, O.

—✦—

Reduced Railroad Rates to Atlanta.—A round trip of one fare plus 25 cents has been secured in all territory South of the Potomac and Ohio and East of the Mississippi.

In all other territory the rate of one and one-third fare on the certificate plan has been granted. Delegates will pay full fare going to Atlanta taking a receipt therefor which will entitle a return at one-third rate. Tickets will be on sale accommodating those who attend the Faculties and Examiners Association as well as the National Dental Association. The return trip date from Atlanta is September 25th.

The Ocean Steamship Co. will, on September 11th, sell tickets at one rate plus $5.00 for the round trip from New York and one fare plus $6.00 from Boston.

J. D. Patterson,

*Chairman Executive Com.,*

National Dental Association.

